# 1-(4-Chloro­phen­yl)piperazine-1,4-diium tetra­chlorido­zincate(II) monohydrate

**DOI:** 10.1107/S1600536808016590

**Published:** 2008-06-07

**Authors:** Imen Ben Gharbia, Riadh Kefi, Meher El Glaoui, Erwann Jeanneau, Cherif Ben Nasr

**Affiliations:** a Laboratoire de Chimie des Matériaux, Faculté des Sciences de Bizerte, 7021 Zarzouna, Tunisia; b Universtié Lyon 1, Centre de Diffractométrie Henri Longchambon, 43 boulevard du 11 Novembre 1918, 69622 Villeurbanne Cedex, France

## Abstract

In the crystal structure of the title compound, (C_10_H_15_ClN_2_)[ZnCl_4_]·H_2_O, the Zn atom is coordinated by four Cl atoms in a tetrahedral geometry. The water mol­ecules and the 1-(4-chloro­phen­yl)piperazine-1,4-diium cations inter­act with the [ZnCl_4_]^2−^ anions through O—H⋯Cl, N—H⋯Cl, N—H⋯O and C—H⋯Cl hydrogen bonds (five simple and one bifurcated). Inter­molecular π–π stacking inter­actions are present between adjacent aromatic rings of 1-(4-chloro­phenyl)­piperazine-1,4-diium cations (the centroid–centroid distance is 3.453 Å).

## Related literature

For related literature, see: Ben Gharbia *et al.* (2005 ▶); Guo *et al.* (2007 ▶); Valkonen *et al.* (2006 ▶); Janiak (2000 ▶).
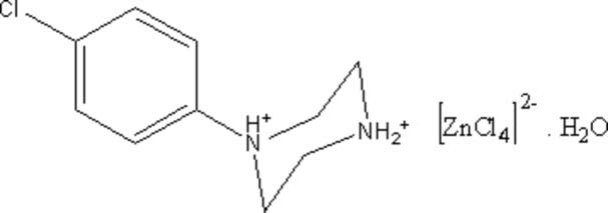


## Experimental

### 

#### Crystal data

(C_10_H_15_ClN_2_)[ZnCl_4_]·H_2_O
*M* *_r_* = 423.90
Monoclinic, 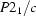

*a* = 7.2036 (2) Å
*b* = 15.1575 (5) Å
*c* = 15.4870 (5) Å
β = 103.012 (2)°
*V* = 1647.58 (9) Å^3^
*Z* = 4
Mo *K*α radiation
μ = 2.29 mm^−1^
*T* = 293 K
0.44 × 0.28 × 0.23 mm

#### Data collection

Nonius KappaCCD diffractometer
Absorption correction: analytical (de Meulenaer & Tompa, 1965 ▶) *T*_min_ = 0.34, *T*_max_ = 0.59
20612 measured reflections
3901 independent reflections
3369 reflections with *I* > 2σ(*I*)
*R*_int_ = 0.085

#### Refinement

*R*[*F* ^2^ > 2σ(*F* ^2^)] = 0.053
*wR*(*F* ^2^) = 0.063
*S* = 0.89
3203 reflections
173 parameters
H-atom parameters constrained
Δρ_max_ = 0.41 e Å^−3^
Δρ_min_ = −0.69 e Å^−3^

### 

Data collection: *COLLECT* (Nonius, 2001 ▶).; cell refinement: *DENZO*/*SCALEPACK* (Otwinowski & Minor, 1997 ▶); data reduction: *DENZO*/*SCALEPACK*; program(s) used to solve structure: *SIR97* (Altomare *et al.*, 1999 ▶); program(s) used to refine structure: *CRYSTALS* (Betteridge *et al.*, 2003 ▶); molecular graphics: *DIAMOND* (Brandenburg, 1998 ▶); software used to prepare material for publication: *CRYSTALS*.

## Supplementary Material

Crystal structure: contains datablocks global, I. DOI: 10.1107/S1600536808016590/bg2190sup1.cif

Structure factors: contains datablocks I. DOI: 10.1107/S1600536808016590/bg2190Isup2.hkl

Additional supplementary materials:  crystallographic information; 3D view; checkCIF report

## Figures and Tables

**Table 1:** Selected bond lengths (Å)

Zn1—Cl1	2.3036 (11)
Zn1—Cl2	2.2937 (11)
Zn1—Cl3	2.2495 (13)
Zn1—Cl4	2.2420 (12)

**Table 2:** Hydrogen-bond geometry (Å, °)

*D*—H⋯*A*	*D*—H	H⋯*A*	*D*⋯*A*	*D*—H⋯*A*
N1—H15⋯O1	0.89	1.86	2.742 (6)	169
N2—H16⋯Cl4^i^	0.89	2.53	3.249 (4)	137
N2—H16⋯Cl2^i^	0.89	2.77	3.352 (3)	123
N2—H17⋯Cl1	0.89	2.42	3.261 (5)	156
O1—H1⋯Cl2^ii^	0.81	2.61	3.342 (3)	149
O1—H2⋯Cl3^iii^	0.82	2.52	3.258 (4)	149
C5—H5⋯Cl4^iv^	0.93	2.76	3.686 (5)	168

Symmetry codes: (i) 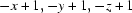; (ii) 
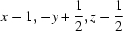
; (iii) 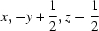; (iv) 
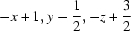
.

**Fig. 1.**
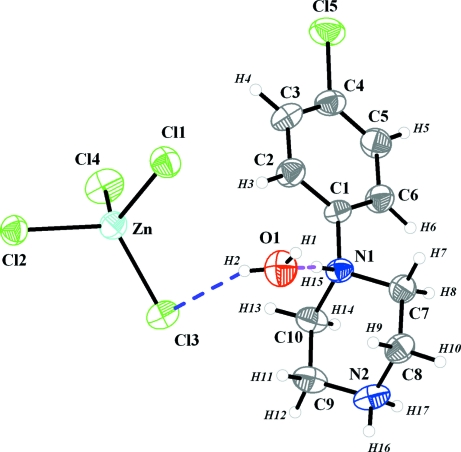
A view of (I), showing 40% probability displacement ellipsoids and arbitrary spheres for the H atoms.

**Fig. 2.**
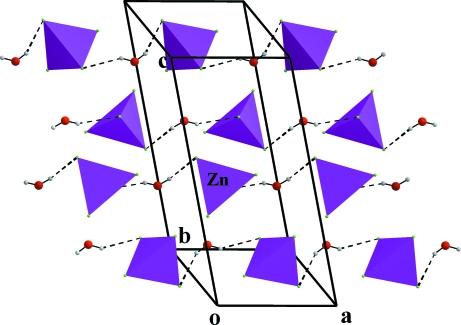
A stereoview of part of the crystal structure showing the formation of (100) chains formed by [ZnCl4]2- tetrahedral anions interconnected through the water molecules.

**Fig. 3.**
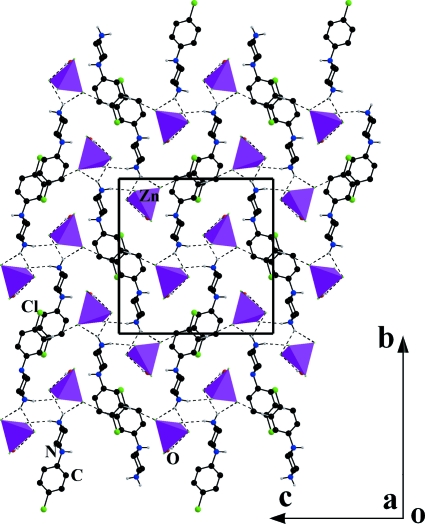
The packing of (I), viewed down the a axis, showing the N—H···Cl, C—H···Cl and O—H···Cl hydrogen bonds between the 1-(4-chlorophenyl)piperazine-1,4-dium cations, water molecules and [ZnCl4]2- anions.

## supplementary crystallographic information

### Comment

The crystal structure of the title compound, (I), (Fig. 1), contains a [ZnCl_4_]^2-^ tetrahedral anion, a 1-(4-chlorophenyl)piperazine-1,4-dium (2+) cation and a water molecule. Fig. 2 shows the atomic arrangement, which can be described as built up by [ZnCl_4_] tetrahedra interconnected through water molecules *via* a O—H···Cl bond to form chains which evolve along the a direction. These [ZnCl_4_].[H_2_O] chains are interconnected into a three-dimensional network by the organic entities through N—H···Cl, C—H···Cl bonds and π-π interactions. Fig.3 shows the way in which two adjacent aromatic rings of the 1-(4-chlorophenyl)piperazine-1,4-dium cations run parallel in the opposite direction and stack each other by turns in a face-to-face mode. The nearest centroid-centroid distance is 3.453 (1) Å, less than 3.8 Å, the maximum value accepted for π-π interactions (Janiak, 2000). Generally, the Zn—Cl bond lengths and Cl—Zn—Cl bond angles in the [ZnCl_4_]^2-^ anion are not equal to one another but vary with the environment around the Cl atoms (Valkonen *et al.*, 2006). In the title compound, the four chlorine atoms of the [ZnCl_4_]^2-^ anion are acting as acceptors of the hydrogen bonds. The bond angles Cl—Zn—Cl vary from 103.37 (5) to 115.30 (5)°, and the bond length of the Zn—Cl lie in the range 2.2420 (12) - 2.3036 (11) Å. Owing to these differences in Zn—Cl bond lengths and Cl—Zn—Cl angles, the coordination geometry of the Zn atom can be described as a slightly distorted tetrahedron (as in Guo *et al.*, 2007). The nearst Zn···Zn intra-chain separation is 7.204 (1) Å, while the distance between adjacent chains is 6.370 (2) Å. Examination of the organic cation geometry shows that the piperazine-1,4-dium ring adopts a typical chair conformation and its geometric parameters [d_av_(C—N) = 1.501 (4) and d_av_(C—C) = 1.508 (4) Å] are in full agreement with those found in phenylpiperazinium tetrachlorozincate (Ben Gharbia *et al.*, 2005).

### Experimental

ZnCl_2_, aqueous 1*M* HCl solution and 1-(4-chlorophenyl)piperazine in a 1:2:1 molar ratio were mixed and dissolved in sufficient ethanol. Crystals of (I) grew as the ethanol evaporated at 293 K over the course of a few days.

### Refinement

The H atoms were all located in a difference map, but those attached to carbon atoms were repositioned geometrically. The H atoms were initially refined with soft restraints on the bond lengths and angles to regularize their geometry (C—H in the range 0.93–0.98, N—H in the range 0.86–0.89 and O—H = 0.82 Å) and *U*_iso_(H) (in the range 1.2–1.5 times *U*_eq_ of the parent atom), after which the positions were refined with riding constraints. The refinement was carried out with 3203 reflections with I>3σ(I). The R factors reported are those calculated for I>2σ(I) (3369 reflections)

### Crystal data

**Table:**

(C_10_H_15_ClN_2_)[ZnCl_4_]·H_2_O	*F*_000_ = 856
*M**_r_* = 423.90	*D*_x_ = 1.709 Mg m^−^^3^
Monoclinic, *P*2_1_/*c*	Mo *K*α radiation λ = 0.71069 Å
Hall symbol: -P 2ybc	Cell parameters from 19642 reflections
*a* = 7.2036 (2) Å	θ = 0.7–27.9º
*b* = 15.1575 (5) Å	µ = 2.29 mm^−^^1^
*c* = 15.4870 (5) Å	*T* = 293 K
β = 103.012 (2)º	Plate, colorless
*V* = 1647.58 (9) Å^3^	0.44 × 0.28 × 0.23 mm
*Z* = 4	

### Data collection

**Table:**

Nonius KappaCCD diffractometer	3369 reflections with *I* > 2σ(*I*)
Monochromator: graphite	*R*_int_ = 0.085
*T* = 293 K	θ_max_ = 28.0º
φ and ω scans	θ_min_ = 1.9º
Absorption correction: analytical(de Meulenaer & Tompa, 1965)	*h* = −9→9
*T*_min_ = 0.34, *T*_max_ = 0.59	*k* = −17→19
20612 measured reflections	*l* = −20→20
3901 independent reflections	

### Refinement

**Table:**

Refinement on *F*	Hydrogen site location: inferred from neighbouring sites
Least-squares matrix: full	H-atom parameters constrained
*R*[*F*^2^ > 2σ(*F*^2^)] = 0.053	*w*eight = 1.0/[1.57 + 1.32*x + 0.866*(2x^2^-1)] * [1-(delta*F*/6*sigma*F*)^2^]^2^where x = *F* /*F*max
*wR*(*F*^2^) = 0.063	(Δ/σ)_max_ = 0.0004
*S* = 0.90	Δρ_max_ = 0.41 e Å^−^^3^
3203 reflections	Δρ_min_ = −0.69 e Å^−^^3^
173 parameters	Extinction correction: none
Primary atom site location: structure-invariant direct methods	

### Fractional atomic coordinates and isotropic or equivalent isotropic displacement parameters (Å^2^)

**Table:**

	*x*	*y*	*z*	*U*_iso_*/*U*_eq_	
Zn1	0.58976 (7)	0.36124 (3)	0.65838 (3)	0.0375	
Cl1	0.29500 (16)	0.42310 (8)	0.60275 (7)	0.0461	
Cl2	0.75399 (15)	0.36476 (7)	0.54758 (6)	0.0415	
Cl3	0.5442 (2)	0.22604 (8)	0.70986 (10)	0.0568	
Cl4	0.7715 (2)	0.44230 (9)	0.76618 (7)	0.0560	
Cl5	0.29277 (18)	−0.13523 (7)	0.48834 (10)	0.0558	
C1	0.2020 (5)	0.1446 (2)	0.4005 (2)	0.0312	
C2	0.2558 (6)	0.0844 (3)	0.3443 (3)	0.0410	
C3	0.2866 (7)	−0.0026 (3)	0.3717 (3)	0.0442	
C4	0.2642 (6)	−0.0255 (3)	0.4557 (3)	0.0404	
C5	0.2183 (7)	0.0359 (3)	0.5131 (3)	0.0441	
C6	0.1869 (7)	0.1230 (3)	0.4851 (3)	0.0399	
C7	−0.0002 (6)	0.2802 (3)	0.3894 (3)	0.0404	
C8	−0.0229 (6)	0.3724 (3)	0.3515 (3)	0.0433	
C9	0.3249 (7)	0.3838 (3)	0.3692 (3)	0.0468	
C10	0.3479 (6)	0.2911 (3)	0.4050 (3)	0.0406	
N1	0.1724 (5)	0.2369 (2)	0.3687 (2)	0.0313	
N2	0.1516 (6)	0.4259 (2)	0.3875 (3)	0.0466	
O1	0.0861 (5)	0.2511 (3)	0.1875 (2)	0.0530	
H1	−0.0199	0.2401	0.1574	0.0730*	
H2	0.1897	0.2453	0.1746	0.0730*	
H3	0.2669	0.1008	0.2869	0.0471*	
H4	0.3213	−0.0443	0.3350	0.0503*	
H5	0.2084	0.0191	0.5702	0.0511*	
H6	0.1563	0.1671	0.5231	0.0473*	
H7	−0.1133	0.2465	0.3632	0.0448*	
H8	0.0133	0.2821	0.4533	0.0447*	
H9	−0.0388	0.3688	0.2866	0.0492*	
H10	−0.1329	0.4027	0.3654	0.0493*	
H11	0.3148	0.3815	0.3047	0.0543*	
H12	0.4375	0.4194	0.3985	0.0543*	
H13	0.4580	0.2643	0.3887	0.0434*	
H14	0.3750	0.2920	0.4706	0.0430*	
H15	0.1556	0.2362	0.3097	0.0410*	
H16	0.1361	0.4792	0.3623	0.0620*	
H17	0.1605	0.4357	0.4450	0.0620*	

### Atomic displacement parameters (Å^2^)

**Table:**

	*U*^11^	*U*^22^	*U*^33^	*U*^12^	*U*^13^	*U*^23^
Zn1	0.0394 (2)	0.0380 (2)	0.0350 (2)	−0.00079 (19)	0.00810 (19)	0.00223 (18)
Cl1	0.0445 (5)	0.0526 (6)	0.0407 (5)	0.0090 (5)	0.0082 (4)	0.0053 (4)
Cl2	0.0449 (5)	0.0428 (5)	0.0392 (5)	−0.0021 (4)	0.0146 (4)	−0.0012 (4)
Cl3	0.0650 (7)	0.0405 (6)	0.0698 (7)	0.0029 (5)	0.0255 (6)	0.0136 (5)
Cl4	0.0704 (7)	0.0562 (7)	0.0350 (5)	−0.0084 (6)	−0.0019 (5)	−0.0027 (4)
Cl5	0.0547 (6)	0.0338 (5)	0.0785 (8)	0.0044 (4)	0.0140 (6)	0.0126 (5)
C1	0.0360 (16)	0.0256 (15)	0.0309 (16)	−0.0002 (14)	0.0055 (14)	−0.0033 (13)
C2	0.048 (2)	0.039 (2)	0.0367 (19)	0.0017 (17)	0.0105 (16)	−0.0033 (16)
C3	0.050 (2)	0.034 (2)	0.047 (2)	0.0069 (17)	0.0085 (18)	−0.0077 (17)
C4	0.0367 (18)	0.0305 (18)	0.051 (2)	0.0021 (15)	0.0034 (16)	0.0003 (16)
C5	0.050 (2)	0.042 (2)	0.041 (2)	0.0025 (18)	0.0112 (17)	0.0089 (17)
C6	0.050 (2)	0.037 (2)	0.0339 (19)	0.0010 (16)	0.0124 (16)	−0.0005 (15)
C7	0.0351 (18)	0.037 (2)	0.051 (2)	0.0050 (15)	0.0128 (16)	0.0027 (17)
C8	0.042 (2)	0.039 (2)	0.049 (2)	0.0095 (17)	0.0085 (17)	0.0014 (17)
C9	0.047 (2)	0.035 (2)	0.054 (2)	−0.0055 (17)	0.0040 (18)	0.0071 (18)
C10	0.0354 (18)	0.033 (2)	0.049 (2)	−0.0031 (15)	0.0012 (16)	0.0045 (16)
N1	0.0391 (16)	0.0263 (14)	0.0274 (13)	0.0008 (12)	0.0055 (12)	0.0007 (11)
N2	0.065 (2)	0.0284 (16)	0.0424 (18)	0.0037 (15)	0.0030 (17)	−0.0020 (13)
O1	0.0519 (18)	0.067 (2)	0.0389 (15)	0.0008 (16)	0.0065 (14)	−0.0023 (14)

### Geometric parameters (Å, °)

**Table:**

Zn1—Cl1	2.3036 (11)	C7—H7	0.970
Zn1—Cl2	2.2937 (11)	C8—N2	1.494 (6)
Zn1—Cl3	2.2495 (13)	C8—H10	0.980
Zn1—Cl4	2.2420 (12)	C8—H9	0.988
Cl5—C4	1.738 (4)	N2—C9	1.485 (7)
C4—C3	1.389 (7)	N2—H16	0.893
C4—C5	1.378 (6)	N2—H17	0.891
C3—C2	1.388 (6)	C9—C10	1.506 (6)
C3—H4	0.922	C9—H12	0.995
C2—C1	1.376 (5)	C9—H11	0.985
C2—H3	0.943	C10—H14	0.990
C1—N1	1.482 (5)	C10—H13	0.973
C1—C6	1.378 (5)	C6—C5	1.392 (6)
N1—C7	1.504 (5)	C6—H6	0.948
N1—C10	1.507 (5)	C5—H5	0.938
N1—H15	0.893	O1—H2	0.820
C7—C8	1.511 (6)	O1—H1	0.818
C7—H8	0.973		
			
Cl1—Zn1—Cl2	107.34 (4)	C7—C8—H10	111.7
Cl1—Zn1—Cl3	107.92 (5)	N2—C8—H10	108.6
Cl2—Zn1—Cl3	115.30 (5)	C7—C8—H9	108.8
Cl1—Zn1—Cl4	112.95 (5)	N2—C8—H9	107.7
Cl2—Zn1—Cl4	103.37 (5)	H10—C8—H9	109.7
Cl3—Zn1—Cl4	110.04 (5)	C8—N2—C9	111.6 (3)
Cl5—C4—C3	118.7 (3)	C8—N2—H16	108.4
Cl5—C4—C5	119.2 (3)	C9—N2—H16	109.4
C3—C4—C5	122.1 (4)	C8—N2—H17	109.2
C4—C3—C2	118.6 (4)	C9—N2—H17	112.9
C4—C3—H4	120.8	H16—N2—H17	105.0
C2—C3—H4	120.6	N2—C9—C10	110.9 (4)
C3—C2—C1	119.0 (4)	N2—C9—H12	108.3
C3—C2—H3	119.7	C10—C9—H12	109.1
C1—C2—H3	121.2	N2—C9—H11	109.6
C2—C1—N1	117.1 (3)	C10—C9—H11	108.5
C2—C1—C6	122.5 (4)	H12—C9—H11	110.5
N1—C1—C6	120.3 (3)	N1—C10—C9	111.0 (3)
C1—N1—C7	113.9 (3)	N1—C10—H14	110.2
C1—N1—C10	110.1 (3)	C9—C10—H14	110.2
C7—N1—C10	110.3 (3)	N1—C10—H13	109.9
C1—N1—H15	107.8	C9—C10—H13	108.7
C7—N1—H15	107.2	H14—C10—H13	106.7
C10—N1—H15	107.3	C1—C6—C5	118.7 (4)
N1—C7—C8	110.1 (3)	C1—C6—H6	120.0
N1—C7—H8	109.4	C5—C6—H6	121.2
C8—C7—H8	110.2	C6—C5—C4	119.0 (4)
N1—C7—H7	109.7	C6—C5—H5	120.7
C8—C7—H7	108.5	C4—C5—H5	120.4
H8—C7—H7	108.9	H2—O1—H1	128.4
C7—C8—N2	110.4 (4)		

### Hydrogen-bond geometry (Å, °)

**Table:**

*D*—H···*A*	*D*—H	H···*A*	*D*···*A*	*D*—H···*A*
N1—H15···O1	0.89	1.86	2.742 (6)	169
N2—H16···Cl4^i^	0.89	2.53	3.249 (4)	137
N2—H16···Cl2^i^	0.89	2.77	3.352 (3)	123
N2—H17···Cl1	0.89	2.42	3.261 (5)	156
O1—H1···Cl2^ii^	0.81	2.61	3.342 (3)	149
O1—H2···Cl3^iii^	0.82	2.52	3.258 (4)	149
C5—H5···Cl4^iv^	0.93	2.76	3.686 (5)	168

Symmetry codes: (i) −*x*+1, −*y*+1, −*z*+1; (ii) *x*−1, −*y*+1/2, *z*−1/2; (iii) *x*, −*y*+1/2, *z*−1/2; (iv) −*x*+1, *y*−1/2, −*z*+3/2.
